# The influence of re-employment on quality of life and self-rated health, a longitudinal study among unemployed persons in the Netherlands

**DOI:** 10.1186/1471-2458-13-503

**Published:** 2013-05-24

**Authors:** Bouwine E Carlier, Merel Schuring, Freek JB Lötters, Bernhard Bakker, Natacha Borgers, Alex Burdorf

**Affiliations:** 1 Department of Public Health, Erasmus MC, PO Box 2040, Rotterdam, CA 30000, The Netherlands; 2 Institute of Health Policy & Management, Erasmus University Rotterdam, Rotterdam, The Netherlands; 3 Inspection Service for Work and Income, Ministry of Social Affairs and Employment, The Hague, The Netherlands

**Keywords:** General health, Quality of life, Re-employment, Unemployment, Longitudinal study

## Abstract

**Background:**

Unemployed persons have a poorer health compared with employed persons and unemployment may cause ill health. The aim of this study was to investigate the effect of re-employment on quality of life and health among unemployed persons on social benefits.

**Methods:**

A prospective study with 18 months follow-up was conducted among unemployed persons (n=4,308) in the Netherlands, receiving either unemployment benefits or social security benefits. Quality of life, self-rated health, and employment status were measured at baseline and every 6 months of follow up with questionnaires. Generalized estimating equations (GEE) modeling was performed to study the influence of re-employment on change in self-rated health and quality of life over time.

**Results:**

In the study population 29% had a less than good quality of life and 17% had a poor self-rated health. Persons who started with paid employment during the follow-up period were more likely to improve towards a good quality of life (OR 1.76) and a good self-rated health (OR 2.88) compared with those persons who remained unemployed. Up to 6 months after re-employment, every month with paid employment, the likelihood of a good quality of life increased (OR 1.12).

**Conclusions:**

Starting with paid employment improves quality of life and self-rated health. This suggests that labour force participation should be considered as an important measure to improve health of unemployed persons. Improving possibilities for unemployed persons to find paid employment will reduce socioeconomic inequalities in health.

## Background

There is ample evidence for socioeconomic differences in health. Persons with a lower education, a lower occupational class, or a lower income die at younger age, and have, within their shorter lives, a higher prevalence of all kinds of health problems [1,2]. Unemployed persons are a specific socioeconomically disadvantaged group. The relationship between unemployment and poor health has been well established, as demonstrated by a higher prevalence of illness and disability [3-6] and a higher mortality among unemployed persons [7]. Two different mechanisms contribute to the poor health among unemployed persons. A poor health increases the probability of leaving the labour force and reduces the possibility of entering paid employment (selection hypothesis) [8-13] and unemployment may cause ill health (causation hypothesis) [13].

Unemployment is deleterious to physical health [6,7,11,13] as well as to mental health [11,13-16]. The causation hypothesis also infers that entering paid employment is beneficial to health, but this mechanism is less well evaluated. Several studies have focused on re-employment and mental health. Various studies found that gaining paid employment improved mental health [16]. Another study found a reduction of distress for unemployed persons who found new jobs [14]. A follow-up study in Norway among unemployed persons showed that re-employment reduced the chance of depression scores to 26% and the chance of anxiety scores to 13% compared to those who were still unemployed [17]. Thus, the positive influence of re-employment on mental health has been consistently demonstrated in several studies.

However, few studies have addressed other aspects of health, such as general health or health-related quality of life. Recently, a Dutch study among unemployed persons living in the City of Rotterdam found a positive effect of re-employment on general health, physical functioning, social functioning, vitality, mental health, bodily pain and role limitations due to emotional or physical problems, with effect sizes ranging from 0.11 to 0.66 [4]. Some studies have broadened the interest towards general quality of life, whereby quality of life is seen as a reflection of the way that persons perceive and react to their health status and all other aspects of their lives. It may be influenced substantially by psychological factors unrelated to health [18,19]. Quality of life generally decreases after unemployment [5,20,21] although one recent study have shown that a considerable number of young adults considered they had attained a better quality of life since unemployment started [22].

Most longitudinal studies on quality of life have examined transitions from employment to unemployment, and there is less agreement about the influence of re-employment on quality of life [23,24]. A German study showed a large drop in quality of life after unemployment and an improvement after re-employment, but quality of life among re-employed persons remained below their original level many years after entering paid employment [23].

In conclusion, the impact of re-employment on mental health seems well-established. There is limited insight into the effect of re-employment on general health and quality of life. The objective of the present study was to determine the effect of re-employment on quality of life and self-rated health among unemployed persons on social benefits in the Netherlands.

## Methods

### Design and study population

A prospective study with 18 months follow-up was conducted among unemployed persons between 18 and 65 years receiving social benefits in the Netherlands. The data collection was part of an internal examination of the quality of the service at The Dutch Employment Centre to beneficiaries of unemployment benefits or social security benefits. Information was obtained on possible barriers for entering paid employment, including ill health, and the role of the social insurance agency in facilitating re-employment. Follow up questionnaires were sent to subjects who had indicated in the former questionnaire to be still unemployed. The Dutch Employment Centre generated a dataset of 70,121 persons, who were on social benefits for at least 6 months in 2006. From this dataset an age-stratified random sample was drawn of 20,847 persons on unemployment benefit (UB) or social security benefit (SSB). Four sequential questionnaires resulted in a 35%, 59%, 40%, and 49% response. Persons with at least two complete questionnaires were included in the study. This resulted in a study sample of 4,308 persons of which 2,604 persons participated two times, 871 persons three times, and 833 persons four times. In order to find out whether the non-respondents differed from the respondents, a sample of 1000 non-respondents was approached with a brief questionnaire. More detailed information on this additional questionnaire is published elsewhere [25].

The two types of social benefits in the Netherlands are determined by a persons’ history of paid work and the duration of the benefit. To be eligible for an unemployment benefit (UB) a Dutch worker must have had paid employment for at least 6 months prior to the benefit application. A social security benefits (SSB) is granted to all unemployed persons who do not have a recent history of paid employment of at least 6 months and do not have sufficient financial means to support themselves. The duration of UB depends on the total number of years worked, with a maximum of 5 years of this benefit. The duration of SSB is not limited in time. Long term unemployed persons and persons who have never worked will receive SSB. At baseline and every 6 months thereafter a questionnaire was sent to the home address, followed by a single reminder after four weeks. Persons who remained unemployed received consecutive questionnaires, whereas those entering paid employment had a maximum follow-up of 6 months after the transition into employment.

The study was conducted by the Inspection of Work & Income of the Ministry of Social Affairs and Unemployment of the Netherlands (IWI) as part of their legal duty. According to Dutch law, approval by a Medical Ethical Committee is not required for a questionnaire survey among adults that does not influence a person’s integrity and does not subject a person to specific procedures or rules of behaviour (information available at http://www.ccmo-online.nl). Participants were fully informed about the purpose and content of the study and the use of data for scientific research. They were informed that filling out the questionnaire would be regarded as provision of informed consent. Participation in the study was voluntary and could not interfere with getting benefits or re-employment activities since no information was transferred between IWI and employment services. IWI complied with the requirements set by the Data Protection Authority, gave permission for the current study, and provided anonymous data to Erasmus MC to carry out the current study. Access to the dataset can only be obtained after permission by IWI.

### Work status

Re-employment was based on self-reports in the questionnaire on having entered paid employment in the past six months. Workers were asked whether they received additional Social Security or Unemployment Benefits. Those who did not receive any social benefit were defined as having paid employment. Persons were also asked the exact date of entering paid employment (see Additional file 1: IWI Questionnaire).

### Health and quality of life

Quality of life was measured with the question ‘How would you rate your life in general in the last six months?’ on a ten point VAS scale [19]. Those reporting less than ‘6’ were defined as having a poor quality of life. Self-rated health was measured by a slightly adjusted question, derived from SF36, “In general, how would you define your health in the last six months?” A five-point scale was used, ranging from ‘very good’, ‘good’, ‘not good/not bad’, ‘poor’, to ‘very poor’ [26]. Those reporting a poor or very poor health were defined as having a poor health and others were classified as good health.

### Sociodemographic measures

Sociodemographic variables, such as age, gender, education, ethnicity, having children and marital status were included in the questionnaire. Persons were divided into three groups according to their highest level of educational attainment. A low educational level was defined as no education, primary school or pre-highschool, an intermediate educational level as highschool or vocational education and a high educational level as higher vocational education or academic degree. Ethnicity was categorized as either native Dutch (both parents were born in the Netherlands) or non-native Dutch according to Statistics Netherlands (CBS 2010). Parenthood was defined as having children under 12 years living at home. Marital status was used to distinguish those persons married or living together from others.

### Data analyses

The exact date of re-employment was unknown for 922 (49%) respondents, for these persons, the re-employment duration was set at the population average of 3 months (imputation). A sensitivity analysis on subjects with complete information on exact month of entering paid employment (n=905) showed similar results based on the observed and imputed values.

In order to study associations at baseline between sociodemographic factors, type of social benefit, with the health status, logistic regression analysis was used. In the first step of the analysis, univariate associations were evaluated. Subsequently, all variables in the univariate analyses with p < 0.20 were investigated in a multivariate analysis using a forward selection technique with significance level of p < 0.05.

Generalized estimating equations (GEE) modeling was performed with quality of life and self-rated health as dependent variables over time. Independent variables were employment status, duration of the employment, time, sex, age, education, ethnic background, parenthood, marital status and type of benefit. This analysis for repeated measurements considered demographic variables as time independent, whereas employment status, duration of the employment, health, and quality of life were time dependent variables. Quality of life and health were dichotomized in order to calculate odds ratios (OR) as measure of association. A simple correlation structure was chosen, assuming a uniform correlation for all possible pairs of variables within persons (exchangeable or compound symmetry). Quality of life and health at baseline were included as independent variables in the models, and, hence an OR above 1 reflects that among those with a transition from unemployment into paid employment health and quality of life improved compared with those without any transition.

In multivariate analyses, differential effects of re-employment on health and quality of life were calculated for gender, age, education, ethnicity and social benefit type. All analyses were performed with the statistical package SPSS-PASW 17 for Windows (Predictive Analytics SoftWare).

## Results

Table 1 shows the sociodemographic characteristics of the study population. Among the unemployed persons 71% had a good quality of life, and 83% had a self-rated good health. Within the study population most persons (81%) were on unemployment benefit whereas a minority (19%) was on social security benefit. Persons on unemployment benefit reported more often a good health and a good quality of life than those on social security benefit, respectively 76% versus 48% (p< 0.05), and 86% versus 67% (p<0.05). Almost half of the unemployed persons (43%) started with paid employment during the follow-up period.

**Table 1:** Socio-demographic characteristics, quality of life, and self-rated health among unemployed persons with benefits at enrolment in the study (n=4308)

**Variables**	**n (%)**
Sex (Woman)	2383 (55.3)
Age	
18–34 years	848 (19.7)
35–44 years	1315 (30.5)
45–54 years	1206 (28.0)
≥ 55 years	939 (21.8)
Education level	
Low	1780 (41.3)
Intermediate	1442 (33.5)
High	1086 (25.2)
Ethnicity (native Dutch)	3439 (79.8)
No children <12 years	3136 (72.8)
Marital status (Living together)	2951 (68.5)
Social security benefit	829 (19.2)
Unemployment benefit	3479 (80.8)
Good quality of life	3045 (70.7)
Self-rated good health	3562 (82.7)

The non-response analysis showed that participation was lower among persons with a lower education, persons on social security benefit, and persons under 35 years of age. An additional questionnaire among non-responders showed no differences in re-employment between responders and non-responders (details about the non-response analysis are published elsewhere [25].

Table 2 shows that younger (< 35 years) high educated persons were more likely to perceive their health as good. In addition, persons who lived together with a partner or received unemployment benefits were also more likely to have a good health. A similar pattern was observed for good quality of life, with the exception of persons of older persons (>55 years) who were more likely to report a good quality of life. There was a moderate correlation between self-rated health and quality of life (Spearman correlation r= 0.42).

**Table 2:** Associations in multivariate logistic regression analyses between sociodemographic measures with self-rated good health and good quality of life among unemployed persons receiving benefits at enrolment in the study (n=4308)

**Variables**	Self-rated good health* OR (95% CI)	Quality of life* OR (95% CI)
Sex (women)	0.87 (0.73–1.04)	1.07 (0.92–1.23)
Age		
18–34 years	1	1
35–44 years	0.77 (0.61–0.98)	0.89 (0.73–1.08)
45–54 years	0.92 (0.71–1.19)	0.97 (0.78–1.19)
55–65 years	0.83 (0.63–1.08)	1.72 (1.35–2.18)
Education level		
Low	1	1
Intermediate	1.20 (0.99–1.45)	1.16 (0.99–1.36)
High	1.37 (1.10–1.70)	1.41 (1.18–1.69)
Ethnicity (Native Dutch)	1.17 (0.96–1.42)	1.59 (1.35–1.88)
No children <12 years	0.80 (0.65–0.99)	0.80 (0.67–0.96)
Marital status (living with partner)	1.50 (1.26–1.79)	1.67 (1.43–1.94)
Social security benefit	1	1
Unemployment benefit	2.68 (2.21–3.26)	2.66 (2.40–3.17)

*Adjusted for sex, age, education, ethnic background, parenthood, marital status and benefit type.

*OR*, Odds ratio; *CI*, Confidence interval.

Table 3 shows that persons who became re-employed were 2.88 times more likely to change from poor to good health compared with those who stayed unemployed (95% CI 2.37–3.50). Up to a maximum of six months after re-employment, every month in paid employment after re-employment, the likelihood of improvement of health increased with 1.05 (95% CI 0.93–1.18).

**Table 3:** The effect of re-employment on the probability to improve from poor to good quality of life and good self-rated health among unemployed persons during 18 months follow-up (n=4308)

	**Good self-rated health**		**Quality of life**	
	Unadjusted model*	Adjusted model**	Unadjusted model*	Adjusted model**
	**OR (95% CI)**	**OR (95% CI)**	**OR (95% CI)**	**OR (95% CI)**
Re-employment transition	3.14 (2.60–3.81)	2.88 (2.37–3.50)	2.21 (2.07–2.36)	1.76 (1.54–2.02)
Time re-employed (months)	1.06 (0.94–1.19)	1.05 (0.93–1.18)	1.12 (1.03–1.23)	1.12 (1.02–1.23)

*Adjusted for time. **Adjusted for time, sex, age, education, ethnic background, parenthood, marital status, and type of benefit. *OR*, Odds ratio; *CI*, Confidence interval.

A similar effect of re-employment on quality of life was observed (Table 3). Re-employed persons were 1.76 times more likely to change from poor to good quality of life (95% CI 1.54–2.02) compared with persons who continued to be unemployed. The duration of being re-employment was also positively associated with quality of life, increasing the likelihood of transition from poor to good quality of life with 1.12 (95% CI 1.02–1.23) with each month.

Among re-employed persons, 60% improved, 40% did not change, and 4% worsened in self-rated health after the employment transition. Among persons who continued to be unemployed, 39% improved, 61% did not change and 9% worsened in self-rated health. For quality of life similar patterns were observed. Among re-employed persons 37% improved, 63% did not change and 8% worsened in quality of life, whereas persons who continued to be unemployed 23% improved, 77% did not change and 8% improved.

The beneficial effect of re-employment on health was more profound among men (OR 3.65 95% CI 2.60–5.12) than among women (OR 2.10 95% CI 1.62–2.71) (Table 4). The positive effect of re-employment on self-rated health and quality of life decreased with increasing age. In addition, among native Dutch persons (OR 4.01 95% CI 3.00–5.14) the increase in health was larger compared to non-native Dutch persons (OR 2.22 95% CI 1.52–3.22). Educational level of type of benefit did not influence the effect of re-employment on health or quality of life.

**Table 4:** Differential effects of re-employment on the probability to improve from poor to good quality of life and self-rated health among unemployed persons during 18 months follow-up for gender, age, and type of benefit (n=4308)

	Good self-rated health adjusted model** OR (95% CI)	Good quality of life adjusted model** OR (95% CI)
**Total population**	2.88 (2.37–3.50)	1.76 (1.54–2.02)
**Gender**		
Men × Unemployed	1	1
Men × Re-employed	3.65 (2.60–5.12)	1.48 (1.22–1.79)
Women × Unemployed	0.82 (0.70–0.96)	1.17 (1.03–1.33)
Women × Re-employed	2.10 (1.62–2.71)	2.40 (1.97–2.94)
**Age**		
18–34 years × Unemployed	1	1
18–34 years × Re-employed	3.07 (1.98–4.76)	1.96 (1.47 (2.61)
35–44 years × Unemployed	0.72 (0.58–0.90)	0.87 (0.72–1.04)
35–44 years × Re-employed	2.49 (1.71–3.63)	1.68 91.30–2.16)
45–54 years × Un-employed	0.83 (0.65–1.06)	0.93 (0.77–1.12)
45–54 years × Re-employed	2.17 (1.45–3.22)	1.66 (1.26–2.18)
55–65 years × Un-employed	0.98 (0.76–1.27)	1.81 (1.47–2.23)
55–65 years × Re-employed	2.07 (1.24–3.46)	2.00 (1.37–2.91)
**Ethnicity**		
Un-employed × Non-native Dutch	1	1
Employed × Non-native Dutch	2.22(1.52–3.22)	1.44 (1.11–1.88)
Un-employed × Native Dutch	1.28 (1.06–1.50)	1.68 (1.46–1.93)
Employed × Native Dutch	4.01 (3.00–5.14)	3.15 (2.60–3.82)

**Adjusted for time, sex, age, education, ethnic background, parenthood, marital status, and type of benefit. *OR*, Odds ratio; *CI*, Confidence interval.

## Discussion

Re-employment had a positive effect on self-rated health and quality of life. Persons who became re-employed were almost three times more likely to improve from poor to good health and 1.76 times more likely to improve from a poor to good quality of life after entering paid employment, compared with those who continued to be unemployed. For every month in paid employment after re-employment, the likelihood of changing towards a good quality of life increased with 1.12.

The longitudinal design with up to four measurements in one and a half year gives more insight into the change of health before and after entering paid employment. Persons only participated in the study one more time after re-employment, so the maximum follow-up after the employment transition was six months. Previous studies showed the largest change in health in the first months after re-employment transition [4,17]. Therefore, this study gives important information on the effects of re-employment on health and quality of life.

The response of the four sequential waves varied between 35% and 59%. Non-participation and loss-to follow up were more frequent among younger, low educated, non-native persons and persons on social security benefits. The current study showed that the effect of re-employment on health was not influenced by educational level or type of benefit. Re-employment resulted in less health benefits among persons with a non-Dutch origin and more health benefits among younger persons. Therefore, the effect of re-employment on health may be biased by selective loss to follow-up.

The monthly improvement of quality of life after re-employment was higher (OR 1.12, 95% CI 1.02–1.23] than the improvement of self-rated health (OR 1.06). The larger improvement of quality of life is likely to be explained by differences in scale size and precision of the two measures (10-point scale compared to 5-point Likert-scale). The proportion of persons reporting poor health might be too small to improve significantly. A more sensitive instrument measuring general health would have showed the positive health effect of the duration of re-employment more clearly. However, a trend in improvement of health and quality of life is demonstrated.

This study showed a positive association between re-employment and a change of self-rated health after controlling for several important determinants of health. Due to practical and ethical reasons, the effect of re-employment on self-rated health cannot be studied in a randomized controlled trial. Nevertheless, in this study we observed a stable proportion of persons experiencing poor health in the group of prolonged unemployed persons (61%) and this stability is in congruence with earlier studies [13]. Repeated measurements analyses showed that among re-employed persons 60% improved in self-rated health. The improvement of health among re-employed persons compared to the stable trend of health among persons who continued to be unemployed provides evidence for a causal relation between re-employment and changes in health.

The positive change in self-rated health as a consequence of re-employment transition is in accordance with findings from previous studies. Recent studies found that re-employment had a positive effect on physical health [4], limiting illness [5] and mental health [4,8,16,27]. Other studies showed the positive effect of re-employment on psychological symptoms [7], well-being [11,14] and life satisfaction [23]. Only two studies addressed physical health, a Dutch study showed that re-employment positively influenced mental health as well as physical health in a short time window [4] and a Norwegian longitudinal study reported a positive effect of re-employment on somatic symptoms [17].

The current study shows that starting with paid employment positively influences health and quality of life. However, there are differences between native and non-native Dutch person in the effect of re-employment on health and quality of life. Persons from minority ethnic groups may be disadvantaged in terms of pay, working conditions and job status, all factors explaining the relation between employment and health [28]. The positive health effect of becoming employed may be limited to certain employment conditions, for instance the psychosocial quality of the work [29], the number of hours worked and the type of contract, flexible versus permanent [27,30]. In addition, among ethnic minorities in the Netherlands, flex-work is on average about twice as high as among Dutch workers [31]. However, although the non-native re-employed persons show a smaller increase in health compared to the native Dutch re-employed persons, they are still better of than their unemployed counterparts. This is also found by Grun et al. [24], who suggests that job quality only matters to some extent, since there is evidence that persons in poor quality employment are still better off, report a higher life satisfaction, than those who remain unemployed.

The current study showed that the effect of re-employment on self-rated health and quality of life decreased with an increase in age. Several studies have found that the negative effect of unemployment on health is especially large for the younger age groups [32]. Older workers who are approaching retirement may be able to cope better with unemployment compared with the younger workers who will be staying longer in the labour force. Therefore, especially among younger persons the negative effect of unemployment on health can be reversed by re-employment.

## Conclusions

This study shows that persons who became re-employed were three times more likely to have a good health status after the transition into paid employment. These results suggest re-employment has a relatively large effect on general health. Unemployed persons are a specific socioeconomically disadvantaged group with a relatively poor health. Re-employment is an important stimulus for improving health and reducing socioeconomic inequalities in health. Hence, labour force participation should be on the health agendas of many national governments [33-35]. Improving possibilities for unemployed persons to find paid employment will have a positive effect on public health.

## Abbreviations

UB: Unemployment benefit; SSB: Social security benefit; GEE: Generalized estimating equations.

## Competing interests

All authors declare that they have no competing interests.

## Authors’ contribution

FJBL and BB are responsible for the data collection. BEC carried out the analysis, interpreted and reported the study results and drafted the manuscript. FJBL, BB and NB provided content expertise. MS and AB conceived the study, provided content expertise, and critically edited all versions. All authors read and approved the final manuscript.

## Pre-publication history

The pre-publication history for this paper can be accessed here:

http://www.biomedcentral.com/1471-2458/13/503/prepub

## Supplementary Material

Additional file 1 IWI Questionnaire. Click here for file

## References

[B1] Mackenbach JP Stirbu I Roskam AJ Schaap MM Menvielle G Leinsalu M Kunst AE Socioeconomic inequalities in health in 22 European countries N Engl J Med 2008 358 2468 2481 10.1056/NEJMsa0707519 18525043

[B2] Mackenbach JP Health 2005 Inequalities: Europe in Profile. An independent, expert report commissioned by, and published under the auspices of, the UK Presidency of the EU

[B3] Weich S Lewis G Poverty, unemployment, and common mental disorders: population based cohort study BMJ 1998 317 115 119 10.1136/bmj.317.7151.115 9657786 PMC28602

[B4] Schuring M Mackenbach J Voorham T Burdorf A The effect of re-employment on perceived health J Epidemiol Community Health 2011 65 639 644 10.1136/jech.2009.103838 20805192

[B5] Bartley M Sacker A Clarke P Employment status, employment conditions, and limiting illness: prospective evidence from the British household panel survey 1991–2001 J Epidemiol Community Health 2004 58 501 506 10.1136/jech.2003.009878 15143119 PMC1732781

[B6] Mathers CD Schofield DJ The health consequences of unemployment: the evidence Med J Aust 1998 168 178 182 9507716 10.5694/j.1326-5377.1998.tb126776.x

[B7] Roelfs DJ Shor E Davidson KW Schwartz JE Losing life and livelihood: a systematic review and meta-analysis of unemployment and all-cause mortality Soc Sci Med 2011 72 840 854 10.1016/j.socscimed.2011.01.005 21330027 PMC3070776

[B8] Thomas C Benzeval M Stansfeld SA Employment transitions and mental health: an analysis from the British household panel survey J Epidemiol Community Health 2005 59 243 249 10.1136/jech.2004.019778 15709086 PMC1733037

[B9] Van den Berg T Schuring M Avendano M Mackenbach J Burdorf A The impact of ill health on exit from paid employment in Europe among older workers Occup Environ Med 2010 67 845 852 10.1136/oem.2009.051730 20798020

[B10] Schuring M Burdorf L Kunst A Mackenbach J The effects of ill health on entering and maintaining paid employment: evidence in european countries J Epidemiol Community Health 2007 61 597 604 10.1136/jech.2006.047456 17568051 PMC2465755

[B11] Wanberg CR The individual experience of unemployment Annu Rev Psychol 2012 63 369 396 10.1146/annurev-psych-120710-100500 21721936

[B12] Claussen B Bjorndal A Hjort PF Health and re-employment in a two year follow up of long term unemployed J Epidemiol Community Health 1993 47 14 18 10.1136/jech.47.1.14 8436885 PMC1059702

[B13] Janlert U Unemployment as a disease and diseases of the unemployed Scand J Work Environ Health 1997 23 79 83 9456072

[B14] Paul KI Moser K Unemployment impairs mental health: Meta-analyses J Vocat Behav 2009 74 264 282 10.1016/j.jvb.2009.01.001

[B15] Price RH Choi JN Vinokur AD Links in the chain of adversity following job loss: how financial strain and loss of personal control lead to depression, impaired functioning, and poor health J Occup Health Psychol 2002 7 302 312 12396064 10.1037//1076-8998.7.4.302

[B16] Murphy GC Athanasou JA The effect of unemployment on mental health J Occup Organ Psychol 1999 72 83 99 10.1348/096317999166518

[B17] Claussen B Health and re-employment in a five-year follow-up of long-term unemployed Scand J Publ Health 1999 27 94 100 10421716

[B18] Diener E Emmons RA Larsen RJ Griffin S The satisfaction with life scale J Pers Assess 1985 49 71 75 10.1207/s15327752jpa4901_13 16367493

[B19] Muldoon MF Barger SD Flory JD Manuck SB What are quality of life measurements measuring? BMJ 1998 316 542 10.1136/bmj.316.7130.542 9501721 PMC2665651

[B20] Blanc P Why quality of life should matter to occupational health researchers Occup Environ Med 2004 61 571 10.1136/oem.2004.013888 15208371 PMC1740813

[B21] Brereton F Clinch JP Ferreira S Employment and life-satisfaction: insights from ireland Economic Soc Rev 2008 39 207 234

[B22] Axelsson L Andersson I Edén L Ejlertsson G Inequalities of quality of life in unemployed young adults: a population-based questionnaire study Int J Equity Health 2007 6 1 1 10.1186/1475-9276-6-1 17374173 PMC1845165

[B23] Lucas RE Clark AE Georgellis Y Diener ED Unemployment alters the set point for life satisfaction Psychol Sci 2004 15 8 13 10.1111/j.0963-7214.2004.01501002.x 14717825

[B24] Grün C Hauser W Rhein T Is Any Job Better than No Job? Life Satisfaction and Re-employment J Lab Res 2010 31 285 306 10.1007/s12122-010-9093-2

[B25] Lötters F Carlier B Bakker B Borgers N Schuring M Burdorf A The influence of perceived health on labour participation among long term unemployed J Occup Rehabil 2012 11 1 9 10.1007/s10926-012-9398-5 23143748

[B26] Ware JE Jr Sherbourne CD The MOS, 36-item short-form health survey (SF-36). I. Conceptual framework and item selection Med Care 1992 30 473 483 10.1097/00005650-199206000-00002 1593914

[B27] Reine I Novo M Hammarström A Does transition from an unstable labour market position to permanent employment protect mental health? Results from a 14-year follow-up of school-leavers BMC Publ Health 2008 8 159 10.1186/1471-2458-8-159 PMC2409329 18477384

[B28] Cooper H Investigating socio-economic explanations for gender and ethnic inequalities in health Soc Sci Med 2002 54 5 693 706 10.1016/S0277-9536(01)00118-6 11999487

[B29] Butterworth P Leach L Strazdins L Olesen SC Rodgers B Broom DH The psychosocial quality of work determines whether employment has benefits for mental health: results from a longitudinal national household panel survey Occup Environ Med 2011 68 806 812 10.1136/oem.2010.059030 21406384

[B30] Strandh M Different exit routes from unemployment and their impact on mental well-being: the role of the economic situation and the predictability of the life course Work Employment Soc 2000 14 3 459 479

[B31] Veenman J Dagevos JM [Ethnic minorities and labour market flexibilisation] Allochtonen en de flexibilisering van de arbeidsmarkt. Tijdschrift Soc Wetenschappen 1999 42 2 106 121

[B32] Bender KA Economou A Theodossiou I The permanent and temporary impact of the unemployment rate on Mortality 2012 The European Experience. International Labour Review

[B33] Baume F The new public health: an Australian perspective 1998 South Melborne: Oxford University Press

[B34] Ågren Gfolkhälsan Sif. Sweden's new public health policy: National public health objectives for Sweden 2003 Sweden: Swedish National Institute of Public Health

[B35] Black CM Working for a healthier tomorrow: Dame Carol Black's review of the health of Britain's working age population 2008 London: TSO

