# Supplementary material for: The influence of re-employment on quality of life and self-rated health, a longitudinal study among unemployed persons in the Netherlands
Source: BMC Public Health. 2013 May 24;13:503. doi: 10.1186/1471-2458-13-503 (PMC3680011; doi:10.1186/1471-2458-13-503)
Supplement: Additional file 1 — IWI Questionnaire. [file 1471-2458-13-503-S1.rtf]

IWI and this study 

This is a questionnaire of The Inspection of Work & Income (IWI) of the ministry of
Social Affairs and Employment (minSZW). 

Anonymity
Please note all responses are completely anonymous. 

IWI questionnaire 
Age
What is your age? _________
Sex
What is your sex?
·	Male 
·	Female
What is your country of birth?
·	Netherlands
·	Morocco
·	Turkey
·	Suriname
·	Dutch Antilles
·	A country in the European Union
·	other


In which country are your parents born?
	Country of mother	Country of father	
Netherlands			
Morocco 			
Turkey			
Suriname			
Dutch Antilles			
A country in the European Union			
other			

Marital status
Are you:
·	I live alone
·	Married/ living together, I am the breadwinner
·	Married/ living together, I am not the breadwinner
·	Married/ living together, we both earn money 
·	Single parent
·	I live with my parents
·	I live with other people
Do you have children living at home?
·	No
·	Yes, younger than 12 years old
·	Yes,  older than 12 years old
Education completed
What is the highest grade or year of school you completed?
·	no education
·	primary school or pre-high school
·	vocational education
·	high school 
·	higher vocational education 
·	academic degree.
Health
How would you rate your life in general from 1 to 10 in the last six months?  _________


In general, how would you say your health was in the last six months?
·	very good
·	good
·	not good/not bad
·	poor
·	very poor

Employment status
Did you enter a paid job in the last 6 months? 
When did you start your paid job? _________ Date
How many hours do you work?     _________  Hours/week
Social Benefit
What kind of Social Benefit do you receive?
·	Social Security benefit
·	Unemployment benefit
·	other
